# Pyruvate transamination and NAD biosynthesis enable proliferation of succinate dehydrogenase-deficient cells by supporting aerobic glycolysis

**DOI:** 10.1038/s41419-023-05927-5

**Published:** 2023-07-06

**Authors:** Luisa Ricci, Federico Uchenna Stanley, Tanja Eberhart, Francesco Mainini, David Sumpton, Simone Cardaci

**Affiliations:** 1 Cancer Metabolism Unit, Division of Genetics and Cell Biology, IRCCS San Raffaele Scientific Institute, 20132 Milan, Italy; 2 CRUK Beatson Institute, Glasgow, UK

**Keywords:** Cancer metabolism, Cell biology

## Abstract

Succinate dehydrogenase (SDH) is the mitochondrial enzyme converting succinate to fumarate in the tricarboxylic acid (TCA) cycle. SDH acts as a tumor suppressor with germline loss-of-function mutations in its encoding genes predisposing to aggressive familial neuroendocrine and renal cancer syndromes. Lack of SDH activity disrupts the TCA cycle, imposes Warburg-like bioenergetic features, and commits cells to rely on pyruvate carboxylation for anabolic needs. However, the spectrum of metabolic adaptations enabling SDH-deficient tumors to cope with a dysfunctional TCA cycle remains largely unresolved. By using previously characterized *Sdhb*-deleted kidney mouse cells, here we found that SDH deficiency commits cells to rely on mitochondrial glutamate-pyruvate transaminase (GPT2) activity for proliferation. We showed that GPT2-dependent alanine biosynthesis is crucial to sustain reductive carboxylation of glutamine, thereby circumventing the TCA cycle truncation determined by SDH loss. By driving the reductive TCA cycle anaplerosis, GPT2 activity fuels a metabolic circuit maintaining a favorable intracellular NAD^+^ pool to enable glycolysis, thus meeting the energetic demands of SDH-deficient cells. As a metabolic syllogism, SDH deficiency confers sensitivity to NAD^+^ depletion achieved by pharmacological inhibition of nicotinamide phosphoribosyltransferase (NAMPT), the rate-limiting enzyme of the NAD^+^ salvage pathway. Beyond identifying an epistatic functional relationship between two metabolic genes in the control of SDH-deficient cell fitness, this study disclosed a metabolic strategy to increase the sensitivity of tumors to interventions limiting NAD availability.

## Introduction

Succinate dehydrogenase (SDH) is a heterotetrameric enzymatic complex catalyzing the oxidation of succinate to fumarate in the tricarboxylic acid (TCA) cycle, the core mitochondrial metabolic pathway for the generation of ATP and several biosynthetic precursors [1]. SDH is the first discovered TCA cycle enzyme with tumor suppressor functions [2]. Heterozygous germline loss-of-function mutations in any human SDH genes (*SDHA*, *SDHB*, *SDHC*, *SDHD*, referred to as *SDHx*), or the SDH complex assembly factor (SDHAF2), are associated with susceptibility to develop familial forms of aggressive malignancies with few therapeutic options and poor prognosis such as paragangliomas and pheochromocytomas, gastrointestinal stromal tumors as well as renal cell carcinoma not associated with a recognized syndrome [2**–**16]. Loss of heterozygosity is frequently detected in *SDHx*-mutated malignancies. This leads to the complete impairment of SDH activity and dysfunction of the TCA cycle in transformed cells, resulting in the consequent accumulation of succinate, an oncometabolite reprogramming gene expression in cancer by activating hypoxia-inducible factors and inhibiting α-ketoglutarate-dependent histone and DNA demethylases [17**–**19].

Alterations in the acquisition and metabolism of nutrients, as well as disposal of waste products, are recognized as hallmarks of cancer development [20]. Mutations in oncogenes and tumor suppressor genes promote complex metabolic reprogramming in cancer cells through changes in the regulation of expression and activity of enzymes and small-molecule transporters. These adaptive changes enable cancer cells to meet the combined biomass and energy demands imposed by uncontrolled cell growth and to invade other tissues where blood supply, and the accessibility to oxygen and other nutrients that comes with it, becomes scarce [21, 22]. How SDH-deficient tumors cope with a dysfunctional TCA cycle to meet metabolic needs remains largely unresolved. Identifying and targeting metabolic functions essential for the growth of SDH-deficient tumors, but dispensable in normal cells, might reveal cancer liabilities exploitable for clinical benefit. By using *Sdhb*-ablated immortalized kidney epithelial mouse cells, we identified major nutritional constraints and metabolic adaptations to SDH loss [23]. We found that lack of SDH activity impairs mitochondrial oxygen consumption and commits cells to rely on aerobic glycolysis for their energetic needs. Maximal glycolytic flux is sustained by the consumption of extracellular pyruvate, whose deprivation impairs the proliferation of SDH-deficient cells, with no effects on normal ones [23]. Also, SDH loss induces a complete truncation of the TCA cycle disabling anaplerotic oxidative metabolism of glutamine for replenishing its intermediates used in biosynthetic reactions. Such metabolic alteration forces SDH-null cells to depend on pyruvate carboxylase to generate oxaloacetate, required for maintaining the aspartate pool. In line, suppression of pyruvate carboxylation selectively reduces the proliferation and tumorigenic capacity of SDH-deficient cells, sparing SDH-proficient counterparts [23].

As the TCA cycle is a metabolic pathway fundamental for cell homeostasis, its impairment, due to lack of SDH activity, is expected to induce a wide range of essential metabolic adaptations. Here, we show that loss of SDH function imposes dependency on mitochondrial glutamate-pyruvate transaminase (GPT2) [24**]** for cell proliferation. GPT2 activity fuels a metabolic circuit maintaining favorable NAD^+^ availability for glyceraldehyde-3-phopshate dehydrogenase (GAPDH) activity by promoting reductive carboxylation of glutamine, thereby sustaining glycolytic demand in SDH-deficient cells. As a metabolic corollary, SDH deficiency increases the sensitivity of cells to NAD^+^ depletion induced by inhibiting the activity of nicotinamide phosphoribosyltransferase (NAMPT), the enzyme controlling the rate-limiting step in the salvage pathway for NAD biosynthesis [25], required to maintain glycolytic flux and proliferative fitness. Beyond disclosing an epistatic relationship between two enzymes in the regulation of SDH-deficient cell fitness, this study identifies a metabolic condition increasing the anti-tumor effects of orthogonal interventions lowering intracellular NAD availability.

## Results

### SDH deficiency stimulates glutamate-pyruvate transamination in cells

**Fig. 1:**
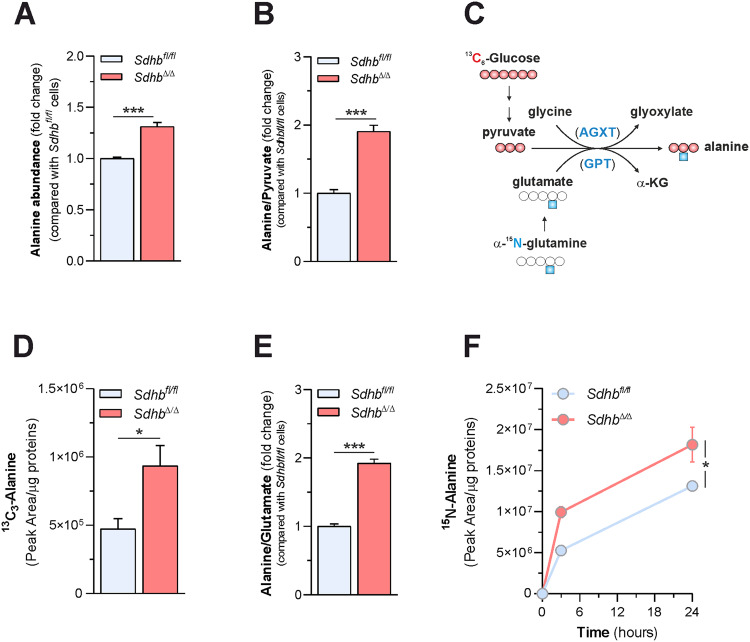
SDH deficiency stimulates glutamate-pyruvate transamination in cells. **A** Measurement of intracellular alanine levels in *Sdhb*^*fl*/*fl*^ and *Sdhb*^*Δ*/*Δ*^ cells. Data were presented as mean ± s.e.m. of *n* = 4 replicates; ***P < 0.001 (two-tailed Student’s *t*-test). **B** Determination of intracellular alanine to pyruvate ratio in *Sdhb*^*fl*/*fl*^ and *Sdhb*^*Δ*/*Δ*^ cells. Data were presented as mean ± s.e.m. of *n* = 4 replicates; ***P < 0.001 (two-tailed Student’s *t*-test). **C** Labeling pattern of the indicated metabolites in cells incubated either with U-^13^C_6_-glucose or α-^15^*N*-glutamine. Red and white circles indicate ^13^C and ^12^C units, respectively. Blue squares indicate ^15^N units. AGXT alanine-glyoxylate aminotransferase, GPT glutamate-pyruvic transaminase. **D** Levels of ^13^C_3_-alanine measured in *Sdhb*^*fl/fl*^ and *Sdhb*^*Δ*/*Δ*^ cells cultured for 24 h in a medium containing U-^13^C_6_-glucose. Data were presented as mean ± s.e.m. of *n* = 3 replicates. *P < 0.05 (two-tailed Student’s *t*-test). **E** Determination of intracellular alanine to glutamate ratio in *Sdhb*^*fl*/*fl*^ and *Sdhb*^*Δ*/*Δ*^ cells. Data were presented as mean ± s.e.m. of *n* = 4 replicates. ***P < 0.001 (two-tailed Student’s *t*-test. **F** Intracellular ^15^N-alanine levels in *Sdhb*^*fl*/*fl*^ and *Sdhb*^*Δ*/*Δ*^ cells incubated with α-^15^N-glutamine for the indicated time. Data were presented as mean ± s.e.m. of *n* = 4 replicates. *P < 0.05 (two-way ANOVA).

Consumption of exogenous pyruvate supports the generation of aspartate, whose biosynthetic rate is not sufficient to meet cell metabolic demands when SDH is lost [23]. Whether SDH loss alters the metabolism of additional amino acids remains largely unresolved. Profiling the exchange rate of metabolites between cells and their culture medium, we previously reported that *Sdhb* ablation results in higher secretion of alanine [23] (Supplementary Fig. 1A), a non-essential amino acid produced by pyruvate transamination. Elaborating on such data, here we show both increased intracellular steady-state levels of alanine (Fig. 1A) and alanine to pyruvate ratio (Fig. 1B) in SDH-deficient (*Sdhb*^*Δ*/*Δ*^) cells compared with SDH-proficient (*Sdhb*^*fl*/*fl*^) counterparts, suggesting an increased alanine generation when SDH activity is lost. As pyruvate is mainly derived from glucose oxidation, we determined directly whether the lack of SDH had an impact on alanine biosynthetic flux by measuring the incorporation of glucose carbons in the alanine pool of cells cultured in the presence of uniformly labeled glucose (U-^13^C_6_-glucose) in the medium (Fig. 1C). Higher levels of ^13^C_3_-alanine were detected in *Sdhb*^*Δ*/*Δ*^ cells compared with *Sdhb*^*fl*/*fl*^ counterparts (Fig. 1D), indicating that glucose carbons are more efficiently channeled into alanine pool when SDH is lost. In mammals, pyruvate can be reversibly transaminated to alanine by accepting an amino group either from glycine, in a reaction catalyzed by alanine-glyoxylate transaminases, or from glutamate, *via* glutamate-pyruvate transaminases (Fig. 1C). Decreased alanine to glycine ratio (Supplementary Fig. 1B) and increased alanine to glutamate proportion (Fig. 1E) were observed in *Sdhb*^*Δ*/*Δ*^ cells with respect to *Sdhb*^*fl*/*fl*^ counterparts, indicating that SDH ablation stimulates alanine biosynthesis by employing glutamate, rather than glycine, as an α-amino donor. By culturing cells in a medium containing U-^13^C_5_-glutamine, we found that the large majority (over 80%) of the intracellular glutamate pool is formed from glutamine deamidation, independent on SDH expression (Supplementary Fig. 1C). Therefore, as a further test of glutamate-pyruvate transamination, cells were cultured in the presence of α-^15^N-glutamine and the kinetics of synthesis of ^15^N-alanine was monitored (Fig. 1C). In agreement with our related findings, ^15^N-labeled alanine was enriched faster in SDH-null cells compared with their controls (Fig. 1F). Collectively, these data demonstrate that SDH deficiency stimulates glucose and glutamine-dependent alanine biosynthesis in cells.

### SDH loss commits cells to rely on GPT2-mediated pyruvate transamination to maintain cell growth

**Fig. 2:**
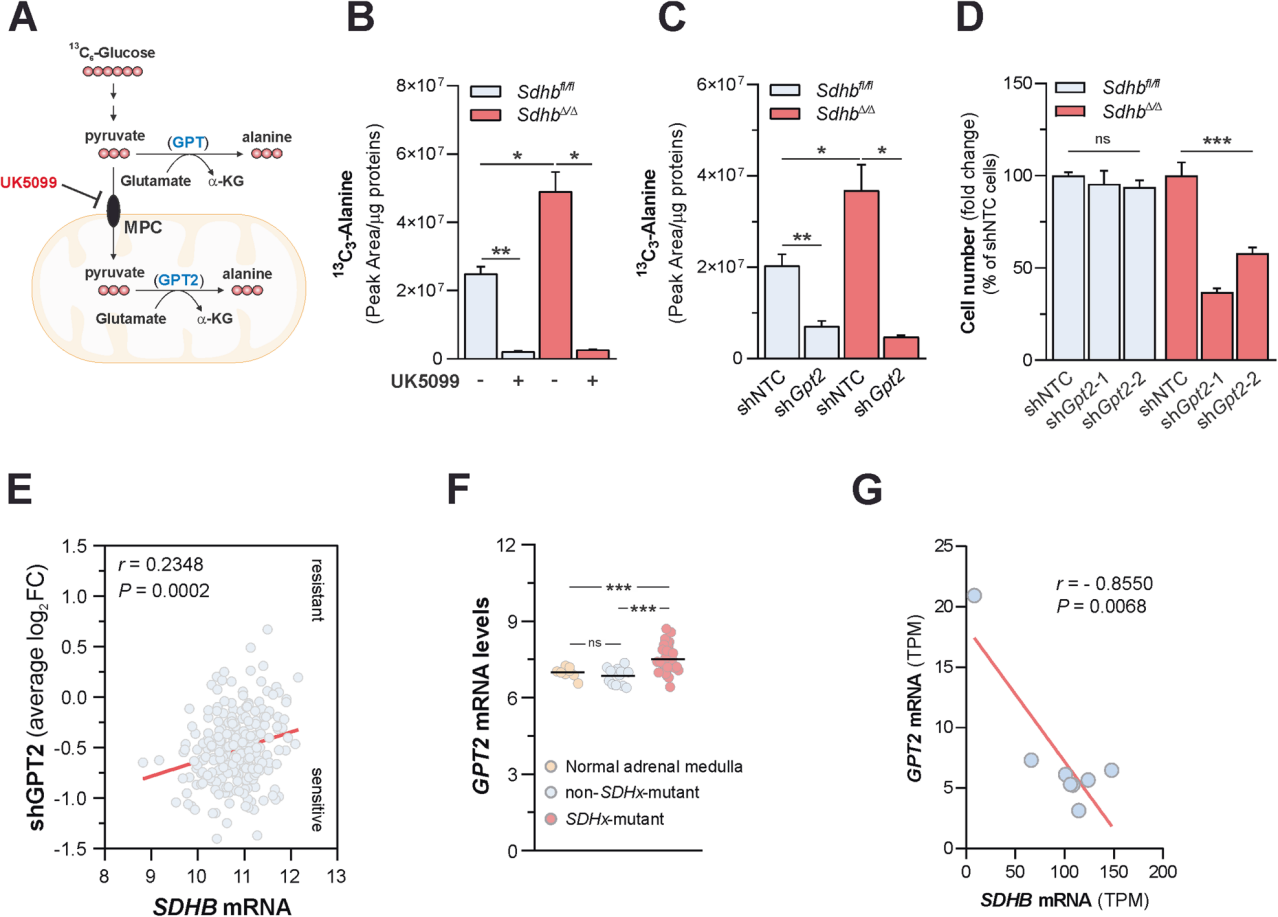
SDH loss commits cells to rely on GPT2-mediated pyruvate transamination to maintain cell growth. **A** Labeling pattern of the indicated metabolites in cells incubated with U-^13^C_6_-glucose. MPC mitochondrial pyruvate carrier, α-KG α-ketoglutarate. In blue, cytosolic (GPT) and mitochondrial (GPT2) glutamate-pyruvate transaminase paralogues. **B** Intracellular levels of ^13^C_3_-alanine in *Sdhb*^*fl*/*fl*^ and *Sdhb*^*Δ*/*Δ*^ cells cultured for 24 h in the presence/absence of the mitochondrial pyruvate carrier (MPC) inhibitor UK5099 (100 µM) in a medium containing U-^13^C_6_-glucose. Data were presented as mean ± s.e.m. of *n* = 3 replicates. *P < 0.05; **P < 0.01 (two-tailed Student’s *t*-test). **C** Intracellular levels of ^13^C_3_-alanine in *Sdhb*^*fl*/*fl*^ and *Sdhb*^*Δ*/*Δ*^ cells infected with lentiviruses expressing either a non-targeting control shRNA (shNTC) or one shRNA sequence targeting *Gpt2* cultured for 24 h in a medium containing U-^13^C_6_-glucose. Data were presented as mean ± s.e.m. of *n* = 4 replicates. *P < 0.05; **P < 0.01 (two-tailed Student’s *t*-test). **D** Number of *Gpt2*-silenced *Sdhb*^*fl*/*fl*^ and *Sdhb*^*Δ*/*Δ*^ cells measured after 96 h of culture. Data were presented as mean ± s.e.m. of *n* = 4 replicates. ***P < 0.001 (one-way ANOVA). **E** Average Log_2_(fold change) of depletion of shRNA sequences targeting *GPT2* (shGPT2) determined in 285 genomically characterized human cancer cell lines retrieved from the Project Achilles plotted in relation to their *SDHB* mRNA levels (microarray) retrieved from Cancer Cell Line Encyclopedia, demonstrating a correlation between sensitivity to *GPT2* silencing and *SDHB* hypoexpression. **F** *GPT2* mRNA expression levels (microarray GSE39716) in human pheochromocytoma and paraganglioma tumor specimens with (*SDHx*-mut, *n* = 32) or without (non-*SDHx*-mut, *n* = 13) mutations in genes encoding for succinate dehydrogenase subunits and normal adrenal medulla tissue (*n* = 8). Each dot indicates one sample, bold black line indicates the mean. ***P < 0.001 (Welch`s-corrected one-way-ANOVA followed by Games-Howell`s multiple comparisons test); ns: not significant. Data were tested for normality. **G** Correlation analysis of *GPT2* and *SDHB* mRNA levels (transcripts per million, TPM) in *n* = 8 human gastrointestinal stromal tumors (GISTs) (RNAseq dataset GSE107447), lacking canonical kinase mutations hand harboring mutations in genes encoding for succinate dehydrogenase subunits. *r* Pearson’s correlation coefficient.

The family of glutamate-pyruvate transaminases consists of two related enzymes encoded by different genes: a cytosolic isoform (GPT, also known as alanine aminotransferase 1) and a paralogue localized into the mitochondrial matrix (GPT2, also named alanine aminotransferase 2) [24]. Therefore, to unravel which isoform primarily accounted for increased alanine biosynthesis when SDH is lost, we measured changes in alanine pools of *Sdhb*^*fl*/*fl*^ and *Sdhb*^*Δ*/*Δ*^ cells, cultured in the presence of U-^13^C_6_-glucose in the medium, in response to pharmacological blockade of pyruvate transport from the cytosol into mitochondria (Fig. 2A). Treatment of cells with UK5099, a small-molecule inhibitor of the mitochondrial pyruvate carrier (MPC) [26], almost totally abolished the contribution of glucose carbons into alanine pool in both cell lines, suggesting that the mitochondrial alanine aminotransferase is the major isoform sustaining glutamate-pyruvate transamination in cells, independently on SDH activity (Fig. 2B). To confirm this indication, we determined the extent to which loss of GPT2 affected alanine biosynthesis in cells. For this aim, *Gpt2* expression was stably silenced by two independent short hairpin RNAs (shRNAs) by lentiviral infection (Supplementary Fig. 2A). Consistent with the effect of MPC inhibition, silencing of *Gpt2* similarly decreased the incorporation of ^13^C carbons derived from U-^13^C_6_-glucose in the alanine pool in both cell types (Fig. 2C). Importantly, despite such results pointed towards GPT2 as an indispensable catalyst of glutamate-pyruvate transamination in cells, regardless of SDH function, suppression of *Gpt2* expression decreased selectively the proliferation of *Sdhb*^*Δ*/*Δ*^ cells, sparing *Sdhb*^*fl*/*fl*^ controls (Fig. 2D), without affecting cell viability (Supplementary Fig. 2B), demonstrating that increased GPT2-mediated alanine biosynthetic flux is a vulnerable adaptation to SDH loss. To understand whether SDH loss imposes dependency on GPT2 for proliferation in cells of human origin, we leveraged genome-scale pooled drop-out shRNA screening data for 285 genomically characterized human cancer cell lines from Project Achilles [27]. For each cell line, averaged sensitivity profiles to shRNAs targeting *GPT* or *GPT2* were correlated to *SDHB* expression data from the Cancer Cell Line Encyclopedia (CCLE) [28]. We observed a significant correlation between the depletion of shRNAs targeting *GPT2* - indicative of the sensitivity of cells to GPT2 loss - and *SDHB* hypoexpression (Fig. 2E). Contrarily, *SDHB* mRNA levels were not associated with the sensitivity of cells to *GPT* silencing (Supplementary Fig. 2C). Similar results were also obtained by tying shRNA screening data to *SDHC* expression (Supplementary Fig. 2D). In all, such results demonstrate that lack of SDH imposes dependency on GPT2 for growth in both mouse and human cells. To extend the translational impact of these findings, we retrieved *GPT* and *GPT2* mRNA levels in publicly-available transcriptomic datasets of human subjects with malignancies harboring loss-of-function mutations in *SDHx* genes. Selective induction of *GPT2* expression was observed in human *SDHx*-mutated pheochromocytoma/paraganglioma (PHEO/PGG) specimens with respect to non-*SDHx*-mutated tumors as well as normal adrenal medulla (Fig. 2F) [29**]**. Similar results were also found by interrogating another related (PHEO/PGG) dataset (Supplementary Fig. 2E) [30]. Contrarily, no major changes in *GPT* expression were found in both studies (Supplementary Fig. 2E, F). Similarly, a significant negative correlation between *SDHB* and *GPT2* mRNA levels were retrieved in human gastrointestinal stromal tumors (GISTs) harboring *SDHx* mutations (Fig. 2G) [31]. On the other hand, no correlation between the expression levels of such genes were found in a different GISTs dataset harboring oncogenic *KIT* or *PDGFRA* mutations (Supplementary Fig. 2G) [32]. In all, such data indicate that decreased *SDHx* expression or activity in tumors driven by oncogenic *SDHx* mutations are associated with the upregulation of *GPT2* mRNA levels. However, the sensitivity of mouse SDHB-deficient cells to suppression of alanine biosynthesis was not determined by changes in either *Gpt2* or *Gpt* expression (Supplementary Fig. 2H, I).

### GPT2 activity drives reductive glutamine carboxylation to sustain glycolysis in SDH-deficient cells

**Fig. 3:**
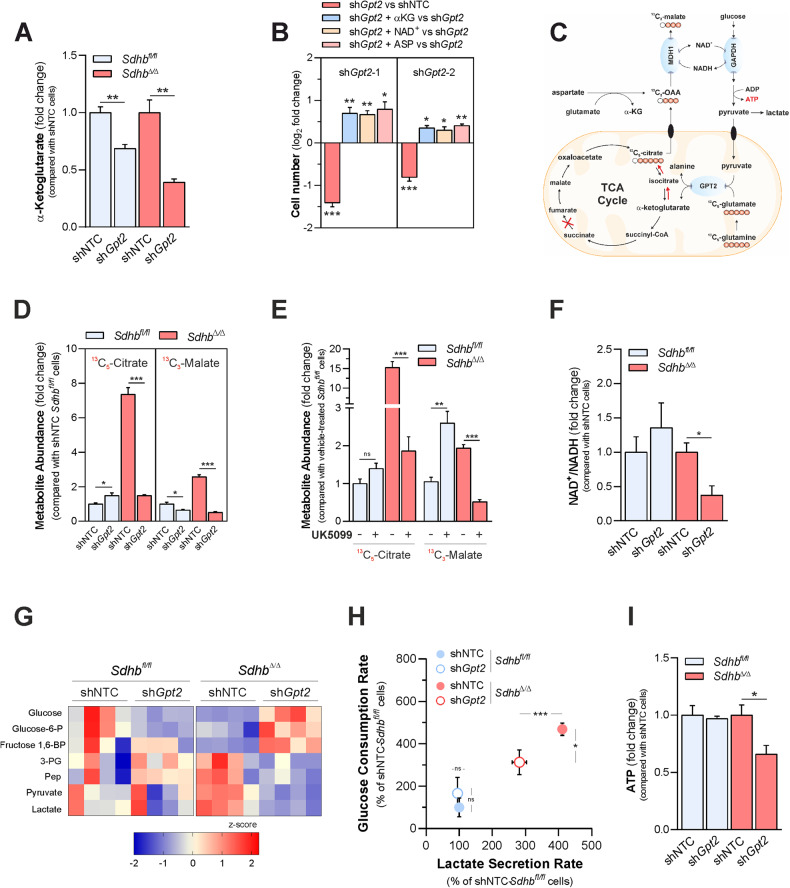
GPT2 activity drives reductive glutamine carboxylation to sustain glycolysis in SDH-deficient cells. **A** Intracellular levels of α-ketoglutarate in *Sdhb*^*fl*/*fl*^ and *Sdhb*^*Δ*/*Δ*^ cells infected with lentiviruses expressing either a non-targeting control shRNA (shNTC) or one shRNA sequence targeting *Gpt2*. Data are presented as mean ± s.e.m. of *n* = 4 replicates. **P < 0.01 (two-tailed Student’s *t*-test). **B** Number of *Gpt2*-silenced *Sdhb*^*fl*/*fl*^ and *Sdhb*^*Δ*/*Δ*^ cells measured after 96 h of culture in the presence/absence of the highest subtoxic concentration of α-ketoglutarate (α-KG, 0.5 mM), 20 mM aspartate (ASP) or 100 uM NAD^+^ in the medium. Data were presented as mean ± s.e.m. of *n* = 4 wells/replicates. *P < 0.05; **P < 0.01; ***P < 0.001 (two-tailed Student’s *t*-test). **C** Labeling pattern of the indicated metabolites in cells incubated with U-^13^C_5_-glutamine. MDH1 malate dehydrogenase 1, GAPDH glyceraldehyde dehydrogenase. **D** Levels of ^13^C_5_-citrate and ^13^C_3_-malate measured in *Gpt2*-silenced *Sdhb*^*fl*/*fl*^ and *Sdhb*^*Δ*/*Δ*^ cells cultured for 24 h in a medium containing U-^13^C_5_-glutamine. Data were presented as mean ± s.e.m. of *n* = 4 replicates. *P < 0.05; ***P < 0.001 (two-tailed Student’s *t*-test). **E** Levels of TCA cycle-derived metabolites ^13^C_5_-citrate and ^13^C_3_-malate in *Sdhb*^*fl*/*fl*^ and *Sdhb*^*Δ*/*Δ*^ cells cultured for 24 h in the presence/absence of 100 µM UK5099 in a medium containing U-^13^C_5_-glutamine. Data were presented as mean ± s.e.m. of *n* = 3 replicates. **P < 0.01; ***P < 0.001 (two-tailed Student’s *t*-test), ns not significant. **F** Determination of intracellular NAD^+^ to NADH ratio in *Gpt2*-silenced *Sdhb*^*fl*/*fl*^ and *Sdhb*^*Δ*/*Δ*^ cells. Data were presented as mean ± s.e.m. of *n* = 3 replicates. *P < 0.01 (two-tailed Student’s *t*-test). **G** Heatmap depicting the intracellular levels of the indicated glycolytic intermediates in *Gpt2*-silenced *Sdhb*^*fl*/*fl*^ and *Sdhb*^*Δ*/*Δ*^ cells. Data were presented as row normalized *Z*-scores of peak area/ug proteins of *n* = 4 replicates. **H** LC-MS-mediated determination of glucose consumption and lactate secretion rates of the indicated cells cultured for 48 h. Data were presented as mean ± s.e.m of *n* = 6 technical replicates. *P < 0.05; ***P < 0.001 (two-tailed Student’s *t*-test). **I** Intracellular levels of ATP in *Gpt2*-silenced *Sdhb*^*fl*/*fl*^ and *Sdhb*^*Δ*/*Δ*^ cells. Data were presented as mean ± s.e.m. of *n* = 4 replicates. *P < 0.05 (two-tailed Student’s *t*-test).

The dependency on GPT2 for proliferation was identified by culturing cells in a DMEM medium, which, like several traditional culture media, lacks alanine in its formulation. However, *Gpt2* loss also selectively affected the proliferative potential of *Sdhb*^*Δ*/*Δ*^ cells cultured in Plasmax [33], a newly developed semi-defined medium containing alanine at the concentration reported for adult human plasma (510 µM) (Supplementary Fig. 2G), suggesting that mechanisms alternative to alanine production accounted for the vulnerability of cells to *Gpt2* silencing when SDH is lost. GPT2-dependent pyruvate transamination is coupled to α-ketoglutarate generation. Consistent with the decrease in alanine biosynthesis, *Gpt2* silencing (Fig. 3A) decreased the steady-state levels of α-ketoglutarate in both in *Sdhb*^*fl*/*fl*^ and *Sdhb*^*Δ*/*Δ*^ cells. Therefore, we envisioned that alterations in α-ketoglutarate production might underlie the impact of suppression of *Gpt2* expression on the proliferative fitness of *Sdhb*^*Δ*/*Δ*^ cells. In line with this hypothesis, the significant rescue of the proliferative capacity of *Gpt2*-silenced *Sdhb*^*Δ*/*Δ*^ cells was observed upon supplementation of the culture medium with supraphysiological levels of α-ketoglutarate (Fig. 3B). In wild-type cells, the oxidative metabolism of glutamine is the major route for replenishing TCA cycle intermediates consumed in anabolic reactions. On the contrary, reductive carboxylation of glutamine is essential for maintaining the proliferation of cancer cells with defective mitochondria [34**–**37]. Therefore, we hypothesized that GPT2-dependent α-ketoglutarate generation might drive the reductive flow of glutamine carbons into the TCA cycle in *Sdhb*^*Δ*/*Δ*^ cells, thus bypassing the oxidative anaplerotic truncation imposed by SDH inactivation [23]. To test this, we measured changes in the abundance of citrate and malate containing ^13^C-glutamine-derived isotopologues generated reductively following *Gpt2* silencing (Fig. 3C). In line with our hypothesis, suppression of *Gpt2* expression decreased the levels of ^13^C_5_-citrate and ^13^C_3_-malate in SDH-deficient cells, eliciting minor impact on SDH-proficient counterparts (Fig. 3D). Similar results were also observed in *Sdhb*^*Δ*/*Δ*^ cells following MPC inhibition (Fig. 3E), demonstrating that GPT2-mediated α-ketoglutarate generation is instrumental to sustain reductive glutamine-dependent TCA cycle anaplerosis when SDH is lost.

Reductive carboxylation of glutamine supports cell proliferation by suppressing mitochondrial-derived reactive oxygen species (ROS) in proliferating cells [35]. However, the impairment of GPT2-dependent reductive TCA cycle anaplerosis did not result in overt oxidative stress in *Sdhb*^*Δ*/*Δ*^ cells, as indicated by the increased ratio between glutathione and glutathione disulfide—which serves as an important indicator of the intracellular redox environment—detected in *Sdhb*^*Δ*/*Δ*^ cells following *Gpt2* silencing (Supplementary Fig. 3A). In agreement, no rescue of proliferative capacity was measured following incubation of *Gpt2*-silenced *Sdhb*^*Δ*/*Δ*^ cells with the antioxidants *N*-acetyl cysteine (NAC) and trolox (Supplementary Fig. 3B). Also, reductive glutamine carboxylation is required to sustain the generation of lipogenic acetyl-coenzyme A, deriving from cleavage of cytosolic citrate, in cells with dysfunctional mitochondria [36]. However, *Gpt2* silencing did not elicit a decline in the steady-state levels of major fatty acids species in *Sdhb*^*Δ*/*Δ*^ cells (Supplementary Fig. 3C).

In cells with mitochondrial dysfunctions, including SDH-deficient cells, oxaloacetate produced *via* reductive carboxylation of glutamine was demonstrated to support glycolytic flux by fueling the activity of cytosolic malate dehydrogenase 1 (MDH1), essential to regenerate NAD^+^ consumed by glyceraldehyde-3-phosphate dehydrogenase (GAPDH) [38]. Therefore, we hypothesized that GPT2 might be required to control NAD^+^ availability for GAPDH activity by driving the reductive metabolic circuit sustaining MDH1 function, thereby promoting bioenergetic fitness when SDH is lost (Fig. 3C). In line with such hypothesis, *Gpt2* silencing decreased NAD^+^/NADH ratio in *Sdhb*^*Δ*/*Δ*^ cells, eliciting no effects in *Sdhb*^*fl*/*fl*^ controls (Fig. 3F).

Such imbalance, not associated to changes in the expression of either *Gapdh* nor *Mdh1* genes (Supplementary Fig. 3D), resulted in a marked accumulation of several detectable glycolytic intermediates upstream of the GAPDH-catalyzed step (glucose, glucose-6-phosphate, fructose 1,6-bisphosphate) and a decrease in the in levels of metabolites generated from 1,3-bisphosphoglycerate processing (3-phosphoglycerate, phosphoenolpyruvate, pyruvate, and lactate) were measured in *Gpt2-*silenced *Sdhb*^*Δ*/*Δ*^ cells, compared with their non-targeting controls, with no consistent changes in SDH-proficient counterparts (Fig. 3G). Consistent with alterations in the steady-state levels of several intracellular glycolytic intermediates, *Gpt2* silencing opposed glucose consumption and lactate secretion of *Sdhb*^*Δ*/*Δ*^ cells without affecting the corresponding exchange rates of *Sdhb*^*fl*/*fl*^ controls (Fig. 3H).

As a consequence of glycolysis impairment, *Gpt2* loss halved ATP levels in *Sdhb*^*Δ*/*Δ*^ cells, without altering the metabolite pool of *Sdhb*^*fl*/*fl*^ controls (Fig. 3I). Aspartate acts as a source of oxaloacetate to fuel MDH1-dependent activity in cells with mitochondrial dysfunctions [38] (Fig. 3C). In agreement with the mechanism here envisioned, supplementation of SDH-deficient cells with aspartate abrogated the effects of *Gpt2*-silencing on NAD^+^ to NADH ratio (Supplementary Fig. 3D), abundances of the glycolytic intermediates (Supplementary Fig. 3E) and ATP levels (Supplementary Fig. 3F). In line with such data, either aspartate or NAD^+^ supplementation significantly rescued the proliferative fitness of SDH-deficient cells, impaired in response to suppression of *Gpt2* expression (Fig. 3B). Taken together, such data demonstrate that SDH loss constrains cells to rely on GPT2-dependent reductive glutamine carboxylation to maintain favorable NAD^+^ availability enabling Warburg-like bioenergetic features.

### A metabolic corollary identifies NAD biosynthesis as a targetable vulnerability to SDH loss

**Fig. 4:**
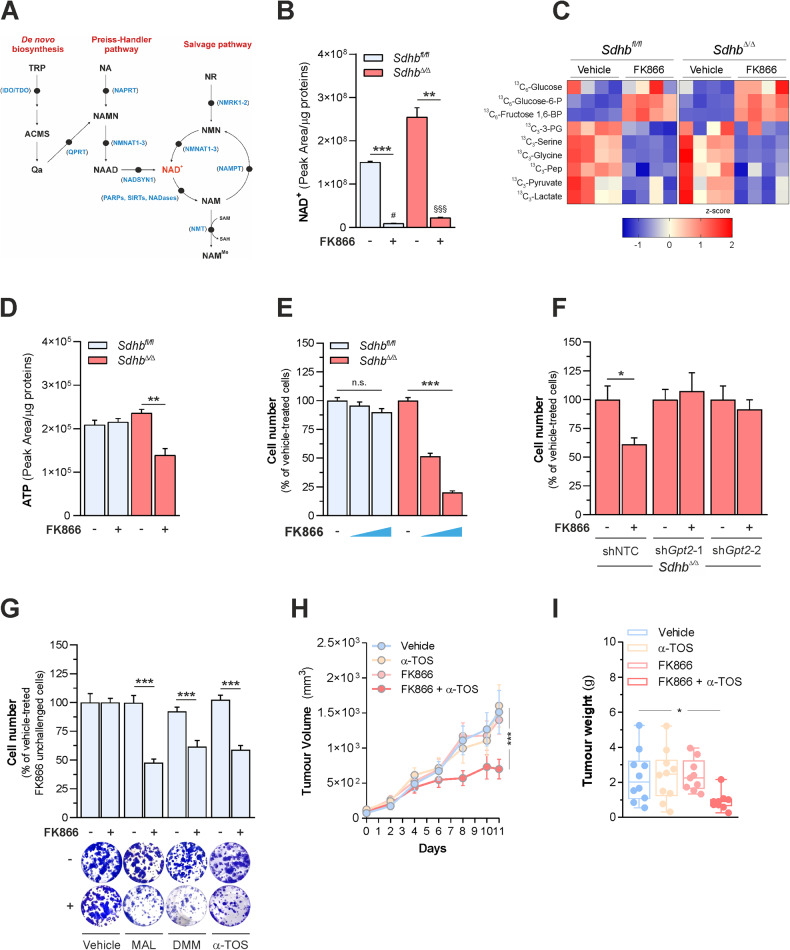
A metabolic corollary identifies NAD biosynthesis as a targetable vulnerability to SDH loss. **A** Overview of NAD biosynthesis pathways. NA nicotinic acid, NAM nicotinamide, NR nicotinamide riboside, NMN nicotinamide mononucleotide, NAAD nicotinic acid adenine dinucleotide, MeNAM *N*-methyl-nicotinamide, NAMN nicotinic acid mononucleotide, ADPR adenosine diphosphate ribose, Qa quinolinic acid, TRP tryptophan, ACMS alpha-amino-beta-carboxymuconate-epsilon-semialdehyde, SAM *S*-adenosyl-methionine, SAH *S*-adenosyl-homocysteine. In blu, the following enzymes: NNMT nicotinamide *N*-methyltransferase, NAMPT nicotinamide phosphoribosyltransferase, NMNAT1-3 nicotinamide nucleotide adenylyltransferase, NAPRT nicotinate phosphoribosyltransferase, NADSYN1 NAD synthetase 1, QPRT quinolinate phosphoribosyltransferase, IDO/TDO indoleamine-2,3-dioxygenase/tryptophan-2,3-dioxygenase, NMRK1-2 nicotinamide riboside kinase 1-2. PARPs poly [ADP-ribose] polymerases, SIRTs sirtuins. **B** NAD^+^ levels in both *Sdhb*^*fl*/*fl*^ and *Sdhb*^*Δ*/*Δ*^ cells cultured for 24 h in the presence/absence of 6 nM FK866 in the medium. Data were presented as mean ± s.e.m. of *n* = 4 replicates. **P < 0.01; ***P < 0.001; # *P* < 0.005 (compared with FK866-untreated *Sdhb*^*fl/fl*^ cells); §§§ *P* < 0.001 (compared with FK866-treated *Sdhb*^*fl*/*fl*^ cells) (two-tailed Student’s *t*-test). **C** Heatmap depicting the intracellular levels of the indicated metabolites in *Sdhb*^*fl*/*fl*^ and *Sdhb*^*Δ*/*Δ*^ cells cultured for 24 h in the presence or absence of 6 nM FK866 in a medium containing U-^13^C_6_-glucose. Data were presented as row normalized *Z*-scores of peak area/ug proteins of *n* = 4 replicates. **D** ATP levels in both *Sdhb*^*fl*/*fl*^ and *Sdhb*^*Δ*/*Δ*^ cells cultured for 48 h in the presence or absence of 6 nM FK866 in a medium containing U-^13^C_6_-glucose. The sum of all ATP isotopologues is reported for clarity. Data were presented as mean ± s.e.m. of *n* = 4 replicates. **P < 0.01 (two-tailed Student’s *t*-test). **E** Number of *Sdhb*^*fl*/*fl*^ and *Sdhb*^*Δ*/*Δ*^ cells measured after 72 h of culture in the presence^*/*^absence of 6–12 nM FK866 in the medium. Data were presented as mean ± s.e.m. of at least *n* = 7 replicates. ***P < 0.001 (one-way ANOVA), n.s. not significant. **F** Number of *Gpt2*-silenced *Sdhb*^*Δ*/*Δ*^ cells measured after 5 days of culture in the presence/absence of 3 nM FK866 in the medium. Data are presented as mean ± s.e.m. of at least *n* = 5 replicates. *P < 0.05 (two-tailed Student’s *t*-test). **G** Effect of pharmacological inhibition of SDH activity on the response of RENCA cells to FK866 treatment. Top, percent of RENCA cell numbers measured after 6 days of culture in the presence/absence of 20 mM malonate (MAL), 3 mM dimethyl malonate (DMM), 5 µM d-α-tocopherol succinate (α-TOS), or 12 nM FK866 in the medium compared to vehicle-treated cells. Data were presented as mean ± s.e.m. of *n* = 6 replicates. ***P < 0.001 (one-way ANOVA followed by Dunnett’s multiple comparisons test, compared with FK866-treated cells). Bottom, representative images of crystal violet^+^ colonies from cells treated as in the top panel. **H** Volumes of tumors originating from the subcutaneous transplant of RENCA cells in BALB/c mice treated daily with FK866 (20 mg/Kg), α-TOS (50 mg/Kg), or the combination of both compounds via intraperitoneal injections for 11 days. ***P < 0.001 (two-way ANOVA). **I** Weight of tumors isolated from mice treated as described in (**H**). *P < 0.05 (one-way ANOVA).

As a metabolic corollary of our findings, we reasoned that SDH loss should impose dependency on NAD^+^ biosynthesis for maintaining bioenergetic fitness. In mammals, the majority of NAD^+^ is synthesized in the salvage pathway from nicotinamide. In this metabolic pathway, nicotinamide is first converted to nicotinamide mononucleotide (NMN), in a reaction catalyzed by the rate-limiting enzyme nicotinamide phosphoribosyltransferase (NAMPT) and then metabolized to NAD^+^ by NMN adenylyltransferases (NMNATs) [25] (Fig. 4A). Therefore, to test the role of SDH on the dependency of cells on NAD^+^ biosynthesis, we measured changes in glycolytic metabolism and proliferative capacities in both *Sdhb*^*fl*/*fl*^ and *Sdhb*^*Δ*/*Δ*^ cells in response to incubation with the pharmacological NAMPT inhibitor FK866. SDH deficiency elicited minor changes in the expression of *Nampt* and other genes encoding for key enzymes of NAD biosynthesis pathways (Supplementary Fig. 4A) and the blockade of NAMPT activity resulted in a significant decline of NAD^+^ levels in both cell lines (Fig. 4B). As a result of limited intracellular NAD^+^ availability, NAMPT inhibition determined an impairment of glycolytic flux independently on SDH function by limiting GAPDH activity, as demonstrated by the accumulation of ^13^C-labeled glycolytic intermediates upstream of glyceraldehyde-3-phosphate and the decreased incorporation of glucose carbons in glycolytic metabolites downstream 1,3-bisphosphoglycerate as well as serine and glycine, amino acids deriving from 3-phospoglycerate processing, in both cell types, in response to FK866 challenge (Fig. 4C). However, in line with the pivotal role of glycolysis in supporting cell bioenergetics when SDH is lost, NAMPT inhibition decreased ATP levels in *Sdhb*^*Δ*/*Δ*^ cells, sparing *Sdhb*^*fl*/*fl*^ controls (Fig. 4D). As a consequence of such bioenergetic impairment, blockade of NAMPT activity decreased preferentially proliferation of SDH-deficient cells, eliciting minor effects on SDH-proficient counterparts (Fig. 4E). Importantly, the effects of FK866 treatment on proliferation of *Sdhb*^*Δ*/*Δ*^ cells were rescued by both NAD^+^ and NMN treatments (Supplementary Fig. 4B), indicating that decreased NAD availability limits proliferation when SDH is ablated. Importantly, as loss of either GPT2 or NAMPT activity is sufficient to affect the bioenergetic fitness of *Sdhb*^*Δ*/*Δ*^ cells by impairing glycolysis, we hypothesized that SDH deficiency should impose an epistatic relation between such enzymes in the control of cell proliferation. In line with this, *Gpt2* silencing abolished the impact of NAMPT inhibition on the proliferation of SDH-deficient cells **(**Fig. 4F), without altering the proliferative capacity of SDH-proficient counterparts (Supplementary Fig. 4C).

NAMPT was identified as a therapeutic target against malignancies [39**–**41]. Therefore, leveraging on the higher sensitivity of SDH-deficient cells to the impairment of NAD biosynthesis, we envisioned that chemical inhibition of SDH activity should potentiate the anti-tumor effects of FK866. To test this hypothesis, we employed RENCA cells, representing the sole available murine renal cell carcinoma cell line tumorigenic in syngeneic immunocompetent (BALB/c) mice. Either CRISPR/Cas9-mediated ablation of *Sdhb* (Supplementary Fig. 4D, E) or pharmacological blockade of SDH activity, achieved by using a panel of small-molecule compounds inhibiting SDH complex (malonate [42], dimethyl malonate [43], d-α-tocopherol succinate (α-TOS) [44]), enhanced the sensitivity of RENCA cells to FK866 challenge in vitro (Fig. 4G). Prompted by these data, we tested the combination of 50 mg/Kg α-TOS and 20 mg/Kg FK866 on the growth of RENCA cells injected subcutaneously in mice. The dose of FK866 selected was effective in decreasing NAD^+^ levels in the originating tumors (Supplementary Fig. 4G). Importantly, although α-TOS and FK866 had no anti-tumor effects when used alone, they significantly decreased volumes (Fig. 4H) and weights (Fig. 4I) of tumors, without no overt effects on normal host behavior and health (data not shown). Moreover, a qualitative decline of Ki-67 immunostaining and a complementary appearance of cleaved caspase-3 positive areas were detected in representative tumors isolated from mice co-treated with FK866 and α-TOS compared with the other three groups (Supplementary Fig. 4H), indicating that the anti-tumor effects observed were associated with both a decrease in proliferation and induction of apoptosis of cancer cells in vivo. Collectively, beyond identifying a targetable metabolic vulnerability in SDH-deficient cells, these data defined a metabolic strategy to enhance response to interventions blocking NAD biosynthesis in tumors.

## Discussion

Oncogenic lesions shape metabolic fluxes in malignant cells, enabling nutrient utilization to meet both anabolic and energy demands imposed by continuous mitotic divisions [45]. TCA cycle truncation resulting from SDH loss was shown to impose dependency on pyruvate carboxylase activity for aspartate generation, thereby creating a metabolic vulnerability exploitable for therapeutic gain [23]. Here, we primarily demonstrate that SDH deficiency commits cells to synthesize alanine *via* the mitochondrial glutamate-pyruvate transaminase GPT2 for cell growth by sustaining the MDH1-dependent metabolic circuit, driven by reductive carboxylation of glutamine, enabling glycolytic flux.

Alanine is a non-essential amino acid synthesized in mammals by transamination of pyruvate from glycine in a reaction catalyzed by alanine-glyoxylate transaminases, or from glutamate, *via* cytosolic (GPT) or mitochondrial (GPT2) glutamate-pyruvate transaminase paralogues [24]. By adopting stable isotope labeling approaches, employing α-^15^*N*-glutamine or ^13^C_6_-glucose as metabolic tracers, we demonstrated that SDH deficiency stimulates glutamate-dependent transamination of glucose-derived pyruvate in cells. Furthermore, by blocking pyruvate internalization in mitochondria, through pharmacological inhibition of the mitochondrial pyruvate carrier, and by silencing *Gpt2* expression, we provide evidence for a primary role of the mitochondrial GPT isoform in sustaining pyruvate transamination in cells, independently of SDH function. Importantly, despite GPT2 act as a non-replaceable catalyst of glutamate-pyruvate transamination, suppression of *Gpt2* expression was sufficient to decrease the selectively proliferative potential of SDH-deficient cells, demonstrating that increased alanine biosynthetic flux is a vulnerable adaptation to SDH loss. Our results also indicate a direct link between *Gpt2* transcriptional levels and either mutational inactivation of *SDHx* genes or suppression of their expression in human tumors. However, induction of *Gpt2* expression was not required to stimulate alanine biosynthesis in SDH-deficient cells cultured in vitro. Such observation allows us to hypothesize that the augmented alanine biosynthetic rate might result either from changes in post-translational or allosteric modulation of GPT2 activity, or be secondary to the increased uptake of glucose and pyruvate from the extracellular milieu imposed by SDH loss [23].

GPT2-dependent pyruvate transamination is coupled to α-ketoglutarate production. On the basis of this, GPT2 activity is known to support the growth of several cancer types by coupling pyruvate, produced by sustained glycolysis, to TCA cycle anaplerosis through the oxidative metabolism of glutamine, representing the major route for replenishing TCA cycle intermediates consumed in anabolic reactions [46**–**48]. On the contrary, reductive carboxylation of glutamine is essential for maintaining the proliferation of cancer cells with dysfunctional mitochondria [37, 49]. By employing ^13^C_5_-glutamine as a metabolic tracer, we demonstrated that GPT2 activity is instrumental to drive the reductive flow of glutamine carbons into the TCA cycle in SDH-deficient cells, thus bypassing the oxidative anaplerotic blockade of such pathway imposed by SDH dysfunction.

Cytosolic malate dehydrogenase 1 (MDH1) generates NAD^+^ upon reduction of oxaloacetate to malate. In cells with mitochondrial respiratory chain impairment, including the SDH-deficient cells used in this study, oxaloacetate produced *via* reductive carboxylation of glutamine, supports glycolytic flux by sustaining MDH1 activity, thus promoting the regeneration of NAD^+^ consumed by glyceraldehyde-3-phosphate dehydrogenase (GAPDH) [38]. Here, we report that GPT2 activity is required for controlling NAD^+^ availability for the GAPDH-catalyzed step of glycolysis, by driving the reductive metabolic circuit sustaining MDH1 function. Such conclusion finds support from data showing a decreased contribution of reductive glutamine carboxylation to the generation of malate pool, a diminished NAD^+^/NADH ratio, a marked accumulation of several glycolytic intermediates upstream of the GAPDH-catalyzed step, coupled with a decreased abundance of metabolites generated from 1,3-bisphosphoglycerate processing, resulting in a drop in ATP levels in SDH-deficient cells, following *Gpt2* silencing. Importantly, such bioenergetic changes were prevented by supraphysiological supplementation of aspartate, known to act as a source of oxaloacetate fueling MDH1-dependent activity in cells with dysfunctional mitochondrial electron transport chain [38]. Glycolytic ATP produced by the MDH1-dependent NADH shuttling promotes cell migration [38**]**. Also, α-ketoglutarate deriving from GPT2-dependent pyruvate to alanine conversion in breast cancer cells is required to drive collagen-based remodeling of the extracellular matrix in the lung metastatic niche [50]. Therefore, as mutational inactivation of *SDHx* genes is associated with increased tumor aggressiveness and metastatic burden [30, 51], it is reasonable to envision a role for GPT2 in promoting metastatic potential of SDH-deficient tumors, in addition to the direct control of cell proliferative potential.

Beyond identifying GPT2 as an enzyme required for cell growth when SDH is lost, this study identified, as a metabolic corollary of the underlying mechanism of action, a vulnerable dependency of SDH-deficient cells on NAD biosynthesis for maintaining their energetic fitness. In mammals, the majority of NAD^+^ is synthetized in the salvage pathway from the conversion of nicotinamide, produced in NAD^+^-consuming reactions, to NMN in a reaction catalyzed by the nicotinamide phosphoribosyltransferase (NAMPT) enzyme [52]. Prior studies established the blockade of glycolysis at the GAPDH step as the central metabolic basis of NAMPT impairment responsible for cell growth inhibition [53]. Aligning to such findings, here we report that pharmacological inhibition of NAMPT activity by FK866 results in a decreased intracellular NAD^+^ availability limiting GAPDH activity, thereby impairing glycolytic flux, independently on SDH function. However, NAMPT inhibition disturbed the selectively energetic fitness of SDH-deficient cells, which almost exclusively depend on glycolysis for ATP production [23], thus decreasing preferentially their proliferative potential. Moreover, it is worth noting that the impact of NAMPT inhibition on glycolysis and bioenergetics of SDH-deficient cells phenocopied that was observed in response to *Gpt2* suppression. Such metabolic superimposition explains the observed epistatic relation between such enzymes in the control of cell proliferation imposed by SDH loss. It is worth noting that while most intracellular NAD^+^ is recycled *via* the NAMPT-controlled salvage pathway from nicotinamide, mammalian cells can synthesize NAD^+^ also de novo from tryptophan by the kynurenine pathway or from niacin and nicotinic acid by the Preiss‐Handler pathway [52]. Therefore, in addition to NAMPT, our results suggest to envision synthetic-lethal interactions between SDH and enzymes metabolizing nutritional sources enabling de novo NAD biosynthesis.

Other than serving as a hydrogen-transferring cofactor in redox reactions, NAD^+^ acts as a substrate for NAD-consuming enzymes, such as poly (ADP-ribose) polymerases (PARPs) and sirtuins, which sustain genomic stability by promoting DNA repair [52]. Succinate increased as a result of SDH loss, is known to suppress the homologous recombination (HR) DNA-repair pathway required for the resolution of DNA double-strand breaks (DSBs), thus rendering tumor cells vulnerable to synthetic-lethal PARP inhibitors [54**–**56]. Therefore, it is conceivable to speculate that, in addition to the bioenergetic default, alteration of genome stability might account for the enhanced sensitivity of cells to FK866 treatment when SDH is lost.

Perturbation of NAD-dependent biological processes might compromise cancer fitness, creating therapeutic windows for clinical benefit. On the basis of this, NAMPT was identified as a target of intervention and FK866, a drug limiting cancer development in preclinical studies [40, 57]. However, some FK866-centered anticancer clinical trials against melanoma, refractory B-cell chronic lymphocytic leukemia, and cutaneous t-cell lymphoma (ClinicalTrials.gov Identifiers: NCT00432107, NCT00435084, and NCT00431912) were stopped due to minor tumor responses and unfavorable side effect profiles thereby claiming new approaches to enhance the clinical impact of pharmacological NAMPT inhibition and reduce drug toxicity. Anticancer drug efficacy and selectivity might be potentiated by strategies generating vulnerable perturbations in cancer cell metabolism [22, 58–60]. Leveraging on the higher sensitivity of SDH-deficient cells to the impairment of NAD biosynthesis, here we demonstrate that pharmacological inhibition of SDH activity potentiates anticancer properties of FK866 without obvious toxicity in immunocompetent hosts.

In conclusion, this study identified alanine and NAD biosynthetic pathways as two epistatic targets of interventions to decrease energetic and proliferative fitness of SDH-deficient cells and, as a consequence, unraveled a metabolic condition enhancing the anti-tumor effects of pharmacological NAMPT inhibition, thereby paving the way for the clinical translation of a novel and effective drug approach against malignancies.

## Methods

### Cell lines and reagents

*Sdhb*-proficient (*Sdhb*^*fl*/*fl*^) and *Sdhb*-deficient (*Sdhb*^*Δ*/*Δ*^) cells were obtained as previously described [23]. If not otherwise stated, *Sdhb*^*Δ/Δ*^-clone 7 was used throughout this study. HEK293T and RENCA cells were obtained from ATCC. The cells were negative for mycoplasma infection and were maintained at 37 °C in a 5% CO_2_ air atmosphere using 25 mM glucose and 1 mM pyruvate-containing DMEM (21969-035, Gibco, Life Technologies) supplemented with 10% heat-inactivated fetal bovine serum (FBS) and 2 mM glutamine. All cell types were routinely checked for mycoplasma contamination. For cell counting, cells were gently detached using trypsin-EDTA 0.05%, centrifuged at 1500 rpm for 5 min, and the cell pellet was re-suspended in fresh media prior to 0.4% Trypan Blue dye exclusion test executed by the LUNA-II^TM^ Automated Cell Counter (Logos Biosystems). Sources of reagents used in this study are indicated throughout the following sections. All reagents not described here were obtained from Sigma-Aldrich.

### Drug treatments

In vitro treatments were performed supplementing cell media with 0.5 mM α-ketoglutarate 20 mM Aspartate, 0.1 mM NAD^+^, 0.1 mM, 0.5 mM *N*-acetyl cysteine, 0.2 mM trolox, 20 mM malonate, 3 mM dimethyl malonate, 5 uM d-α-tocopherol succinate, for the time indicated in each figure legend. Such compounds were solubilized either in ultrapure H_2_O or DMSO (as per manufacturer instructions) and equal volumes of such solvents were used as vehicle controls. FK866 was solubilized in DMSO and used at concentrations indicated in figure legends.

### Determination of cell number and viability

Cells were plated at 1 × 10^4^ cells per well in 24-well plates. At the specified experimental time, each well was washed once with PBS, trypsinized, re-suspended in 0.4% Trypan Blue dye, and viable cells were counted by the LUNA-IITM Automated Cell Counter (Logos Biosystems).

To determine the impact of *Gpt2* silencing on cell viability, cells were plated at 1 × 10^4^ cells per well in 96-well plates and after 96 h, the activity of l-Lactate dehydrogenase (LDH) was determined colorimetrically in their culture media by using the CyQUANT™ LDH Cytotoxicity Assay (Thermo Fisher Scientific), according to manufacturer instructions. LDH activity in media of cells treated with 1 mM H_2_O_2_ for 96 h was used as a positive control of cytotoxicity.

In experiments using FK866 and inhibitors of SDH activity, cells were pleated at 1 × 10^3^ cells per well in 96-well plates and the number of viable cells was determined by the crystal violet assay. Briefly, cells were washed with PBS, fixed with 4% PFA for 10 min, and stained with a solution of 0.1% crystal violet in 4% methanol. After 10 min, cells were washed with water, crystal violet dye was solubilized with 10% acetic acid, and the absorbance was measured at 570 nm by a Biorad microplate reader.

### CRISPR–Cas9 constructs

Non-targeting control sequence and one single guide RNAs (sgRNA) against exon 4 of *Sdhb* were cloned into LentiCRISPRv2 [61] using the BsmBI restriction enzyme. For each sequence, 3 × 10^5^ RENCA cells were transfected with 1 µg of gRNA using lipofectamine (BRAND - NAME). Twenty-four hours after transfection, the media were replaced and supplemented with 1 µg/ml puromycin for selection. Puromycin-resistant cells were seeded in 96-well plates at the concentration of 0.5 cells/well. Individual clones were collected, and SDHB expression was tested by immunoblotting. The following sgRNA target sequences were used: non-targeting control sgRNA, 5′-GTAGCGAACGTGTCCGGCGT-3′; *Sdhb* sgRNA 5′- ATACTCTGGCGTGCACACGCAGG-3′.

### Metabolite extraction and LC-MS

*Sdhb*^*fl*/*fl*^ and *Sdhb*^*Δ*/*Δ*^ cells were plated in six-well plates in 25 mM glucose and 1 mM pyruvate-containing DMEM (21969-035, Gibco, Life Technologies) supplemented with 10% FBS and 2 mM glutamine at 2 × 10^5^ and 3 × 10^5^ cells per well, respectively. Cells were cultured in the specified conditions for 24 h (if not otherwise indicated) for intracellular metabolite (endo-metabolite) determination. For stable isotopic tracing assays, 24 h after cell plating, media were replaced with (i) DMEM (11966-025, Gibco, Life Technologies), supplemented with 10% FBS, 1 mM pyruvate and 25 mM U-^13^C_6_-glucose for glucose-tracing experiments; (ii) DMEM (21969-035, Gibco, Life Technologies) supplemented with 10% FBS and 4 mM U-^13^C_5_-glutamine was used, for glutamine-tracing experiments. For α-15*N*-glutamine-tracing assays, cells were plated in 25 mM glucose and 4 mM glutamine-containing custom-made DMEM supplemented with 10% FBS with or without 1 mM pyruvate. After 24 h, media were replaced with the same medium containing 2 mM unlabeled glutamine. After 6 h, cells were incubated with 2 mM α-15N-glutamine. Cells were cultured in the specified conditions for 24 h. At the end of the incubations, monolayers were rapidly washed three times with ice-cold PBS and extracted with 600 μl of ice-cold extraction solution, composed of methanol, acetonitrile, and water (5:3:2), for endo-metabolite determination. Alternatively, media were diluted 1:50 in extraction solution for exo-metabolite analyses. Media derived from wells lacking cells but incubated in the same conditions were used as a reference to quantify the exchange rate (consumption/secretion) of exo-metabolites. Culture media and cell extracts were then centrifuged at 16,000×*g* for 30 min at 4 °C and the supernatants were analyzed by liquid chromatography-mass spectrometry (LC-MS) by using a Q Exactive Orbitrap mass spectrometer (Thermo Fisher Scientific) coupled with a Thermo Fisher Scientific Accela HPLC system, as previously described [62**]**. Briefly, the HPLC setup consisted of a ZIC-pHILIC column (SeQuant, 150 mm × 2.1 mm, 5 µm, Merck KGaA) with a ZIC-pHILIC guard column (SeQuant, 20 mm × 2.1 mm) and an initial mobile phase of 20% 20 mM ammonium carbonate, pH 9.4 and 80% acetonitrile. Cell extracts (5 µl) were injected and metabolites were separated over a 15-min linear gradient decreasing the initial acetonitrile content to 20% at a flow rate of 200 μl min^−1^ and holding the column temperature at 45 °C. Metabolites were detected across a mass range of 75–1000 m/z using the Exactive mass spectrometer at a resolution of 25,000 (at 200 m/z), with electrospray ionization and polarity switching to enable both positive and negative ions to be determined in the same run. Lock masses were used and the mass accuracy obtained for all metabolites was below 5 p.p.m. Data were acquired with Thermo LCquan 2.7 (Thermo Fisher Scientific) software. Endo-metabolites were normalized to the protein content in each well determined, at the end of the experiment, using the Lowry assay. The exchange rates were obtained by normalizing the variation in consumption or secretion of each exo-metabolite to the average protein content of each sample from the beginning of the experiment (*t* = 0 h) to its end (*t* = 48 h).

For measurement of NAD^+^ abundance in subcutaneous tumors, frozen tumor were weighed and dissociated in cold extraction solution (40 mg/ml) using ceramic beads and a Precellys homogenizer (Bertin Instruments). After centrifugation (30 min, 16,000×*g*), the supernatants were used for LC-MS analysis as above described.

#### RNA extraction and real-time qPCR

RNA was extracted with ReliaPrep^TM^ RNA Cell Miniprep System (Z6011 Promega) according to the manufacturer’s instructions. For real-time qPCR analysis, 1 μg of total RNA was retro-transcribed into complementary DNA using SuperScript VILO reverse transcriptase (11755-050, Invitrogen). cDNAs were mixed with PowerUp™ SYBR™ Green Master Mix (A25742, Applied Biosystems) according to the manufacturer’s instructions and 200 nM of both forward and reverse primers for detecting mRNA levels of the following mouse genes: *Gpt2* (forward 5′-ACTAAACAGCCCCAGCTTCC-3′; reverse 5′-GCACTGTAAGATCCCAAGCTGT-3′), *Gpt* (forward 5′-AAACTGATGAGCGTGCGGTT-3′; reverse 5′-CCTGCCTCTCTGCTTGAAACT -3′), *Nampt* (forward 5′- TACTGTGGCGGGAATTGCTC-3′; reverse 5′- GCCGTTATGGTACTGTGCTCT-3′), *Mdh1* (forward 5’- TTCTGGACGGTGTCCTGATGGA -3’; reverse 5’- TAGGACAGCCACATCCAGGTCT -3’), *Gapdh* (forward 5’- CATCACTGCCACCCAGAAGACTG -3’; reverse 5’- ATGCCAGTGAGCTTCCCGTTCAG -3’), Nampt (forward 5’- TACTGTGGCGGGAATTGCTC -3’; reverse 5’- GCCGTTATGGTACTGTGCTCT -3’), *Nmnat1* (forward 5’- GTGGAGACTGTGAAGGTGCTC -3’; reverse 5’- GTGAGCTTTGTGGGTAACTGC -3’), *Nmnat2* (forward 5’- GACCGAGACCACAAAGACCC -3’; reverse 5’- CCCTGGCTCTCTCGAACATC -3’), *Nmnat3* (forward 5’- CAAACAGGAAGGTACCAGGTGA -3’; reverse 5’- TCCACCCGAATCCAGTCAGA -3’), *Nmrk1* (forward 5’- GGTAGGCATGAAACGCTCTTG -3’; reverse 5’- CCGTTTGTCACACCACCAA -3’), *Nadsyn1* (forward 5’- AAGTTGCCTCGGTTCTCAGC -3’; reverse 5’- ACCATCCTAATCCGAGCCTTCC -3’), *Qprt* (forward 5’- GTGGAATGTAGCAGCCTGGA -3’; reverse 5’- TGCAGCTCCTCAGGCTTAAA -3’), *Naprt* (forward 5’- AGTTCGAGCTCTTCTTCCGC -3’; reverse 5’- AACTGCACATCTGCATCCCG -3’), *Rps18* (forward 5′-CACTTTTGGGGCCTTCGTG -3′; reverse 5′-GCAAAGGCCCAGAGACTCATT-3′). qPCR was carried out using a CFX Connect Real-Time PCR System (BioRad) followed by melt curve analysis. The relative quantification of each mRNA was carried out with the comparative threshold cycle method using ribosomal protein S18 (*Rps18*) for normalization.

#### Immunoblotting

Cells were washed twice with cold PBS and lysed in radioimmunoprecipitation assay (RIPA) buffer supplemented with protease (cOmplete™ Mini EDTA-free Protease, Roche) and phosphatase (PhosSTOP EASYpack, Roche) inhibitor cocktails. Protein concentration was determined with the BCA Protein Assay Kit (23227, Pierce) using BSA as a standard. Equal amounts of protein were mixed with reducing Laemmli 4X buffer, warmed at 95 °C for 5 min, and loaded on 10% gels for SDS–PAGE. After electrophoretic separation, proteins were blotted onto 0.45 mm nitrocellulose (Amersham Protran), blocked with 10% non-fat milk in phosphate-buffered saline-Tween, and incubated at 4 °C overnight with the following antibodies: anti-GPT2 (rabbit polyclonal, Proteintech 16757-1-AP, 1:1000), anti-NAMPT (mouse monoclonal, Adipogen AG-20A-0034, 1:2000) and anti-β-Actin (mouse monoclonal, Sigma-Aldrich, A5441, 1:10000). Membranes were then washed and incubated with HRP Linked anti-rabbit (NA934V, GE Healthcare) or anti-mouse (NA931V, GE Healthcare) secondary antibodies at 1:10,000 dilution. A chemiluminescent signal was acquired using a ChemiDoc^TM^ MP Imaging System (Biorad) after incubation with Supersignal West Pico PLUS Chemiluminescent Substrate (34580, Thermo Fisher Scientific).

#### shRNA transductions

The lentiviral non-targeted shRNA SCR and shRNA plasmids against *Gpt2* were purchased from Sigma-Aldrich and identified as follows: Gpt2-1 shRNA, TRCN0000119733 (forward 5'-CCGGCGGTATTTCTACAATCCTGAACTCGAGTTCAGGATTGTAGAAATACCGTTTTTG-3'; reverse 5’-AATTCAAAAACGGTATTTCTACAATCCTGAACTCGAGTTCAGGATTGTAGAAATACCG-3’); Gpt2-2 shRNA, TRCN0000119732 (forward 5′-CCGGGCTAGGCATATAATCCAGATACTCGAGTATCTGGATTATATGCCTAGCTTTTTG-3′; reverse 5’-AATTCAAAAAGCTAGGCATATAATCCAGATACTCGAGTATCTGGATTATATGCCTAGC-3’). Lentiviral plasmids were transfected into HEK293T cells together with packaging and envelope plasmids (psPAX2 and *VSV-G*) using a calcium phosphate procedure. Two days after transfection, the growth medium containing lentiviruses was filtered through a 0.45-μm pore filter, mixed with 8 µg/ml Polybrene (H9268, Sigma-Aldrich), and transferred to the recipient cells. HEK293T cells were further cultured in a fresh medium for 24 h. The next day, the infection was repeated as above. After lentivirus infection, cells were selected with 2 μg/ml Puromycin (Sigma-Aldrich, Cat#: P9620) for 48 h. Then, the medium was changed, and cells were plated for further experiments as described above. If not otherwise stated, the sh*Gpt2*-1 sequence was used in the experiments.

### RENCA allografts

RENCA cells were subcutaneously injected into the rear flanks of 10-week-old male syngeneic Balb/c mice, randomized, and allocated to experiment groups. When tumors reached 50 mm^3^ volume, FK866 (20 mg/Kg), and α-TOS (50 mg/Kg)—both dissolved in corn oil—the combination of both compounds or an equal amount of corn oil, as vehicle control, were administered to mice once per day via intraperitoneal injections for 11 days. Tumor lengths (L) and widths (W) of sagittal sections were measured twice per week using a manual calliper, and tumor volumes were calculated with the formula tumor volume = 0.5 × L × W^2^. No statistical method was used to predetermine the sample size. The investigators were not blinded to allocation during experiments and outcome assessment.

#### Immunohistochemistry

Formalin-fixed (HT501128, Sigma-Aldrich) paraffin-embedded sections of subcutaneous tumors were dewaxed and hydrated through a graded decrease alcohol series. For immunohistochemistry (IHC), slides were immunostained with the Automatic Leica BOND RX system (Leica Microsystems GmbH, Wetzlar, Germany). Antigen-retrieval was performed using sodium citrate buffer at 100 °C. Primary antibodies anti-Ki-67 (dilution 1/200; Ki-67 (D3B5) #12202, Cell Signaling Technology, Inc.) and anti-cleaved caspase-3 (1/400; Cleaved Caspase-3 (Asp175), #9661, Cell Signaling Technology, Inc.) were developed with Bond Polymer Refine Detection (Leica, DS9800). Bright-field images were acquired with an Aperio AT2 digital scanner (Leica Biosystems) and Aperio ImageScope software (v12.4.3.5008, Leica Biosystem).

#### Human datasets

The microarray datasets E-MTAB-733 [30] and GSE39716 [29] integrate RMA normalized mRNA expression values from pheochromocytomas and paragangliomas with different germline mutations (VHL, RET, NF1, SDHA, SDHB, SDHC, SDHD, or TMEM127). The RNAseq datasets GSE107447 [31] contains transcriptomic profiles of SDH-deficient gastrointestinal stromal tumors. The microarray dataset GSE136755 [32] comprises signal intensity normalized mRNA expression values from gastrointestinal stromal tumors with oncogenic KIT or PDGFRA mutations.

#### Ethical approval of animal studies

Balb/c mice were obtained from Charles River. Animals were housed in individually ventilated cages in a barrier facility proactive in environmental enrichment under specific pathogen-free conditions in line with European Union regulations. All experimental animal procedures were approved by the Institutional Animal Committee of San Raffaele Scientific Institute.

### Statistics and reproducibility

For each experiment, the sample size was chosen on the basis of similar experimental approaches reported in the literature. If not otherwise stated in each figure caption, data were shown as mean ± SEM. Details of the group size (“*n*”) are provided in each figure legend. Two-tailed Student *t*-tests (with Welch’s correction when unequal variances between two experimental groups were computed), one-way Anova, two-way Anova, and Pearson’s correlation were calculated by GraphPad Prism 8 software.

## Supplementary information

Supplementary Material

Supplementary Figure 1

Supplementary Figure 2

Supplementary Figure 3

Supplementary Figure 4

Supplementary Material - Uncropped Western Blots

Reproducibility checklist

## Data Availability

All data that support the findings in this study are stored at the IRCCS San Raffaele Scientific Institute and are available from the corresponding author upon reasonable request.
