# Supplementary material for: Pyruvate transamination and NAD biosynthesis enable proliferation of succinate dehydrogenase-deficient cells by supporting aerobic glycolysis
Source: Cell Death Dis. 2023 Jul 6;14(7):403. doi: 10.1038/s41419-023-05927-5 (PMC10326256; doi:10.1038/s41419-023-05927-5)
Supplement: Supplementary file 1 — Supplementary Material [file 41419_2023_5927_MOESM1_ESM.docx]

**Supplementary Information**

**Pyruvate transamination and NAD biosynthesis enable proliferation of succinate dehydrogenase-deficient cells by supporting aerobic glycolysis**

Ricci L. et al.

**Supplementary Figure 1. SDH deficiency stimulates glutamate-pyruvate transamination in cells.** (**A**) Determination of alanine secretion rate in the medium of cells cultured for 48h with U-^13^C_6_-glucose. The sum of all isotopologues is reported for clarity. Data were extracted from reference n.23 (Supplementary Fig.3A) and presented as mean±s.e.m of n=18 replicates. **P*<0.05 (one-way Anova followed by Dunnett's multiple comparisons test, compared with *Sdhb^fl^*^/^*^fl^* cells). (**B**) Determination of intracellular alanine to glycine ratio in *Sdhb^fl^*^/^*^fl^* and *Sdhb^Δ^*^/^*^Δ^* cells. Data are presented as mean ± s.e.m. of *n* = 4 replicates.  ***P*<0.01 (two-tailed Student’s *t*-test). (**C**) Relative abundance of glutamate formed by deamidation of glutamine (^13^C_5_-glutamate) in *Sdhb^fl^*^/^*^fl^* and *Sdhb^Δ^*^/^*^Δ^* cells cultured for 24h in medium containing U-^13^C_5_-glutamine. Data are presented as mean ± s.e.m. of *n* = 4 wells.

**Supplementary Figure 2. SDH loss commits cells to rely on GPT2-mediated pyruvate transamination to maintain cell growth.** (**A**) qPCR analysis of *Gpt2* mRNA levels in *Sdhb^fl^*^/^*^fl^* and *Sdhb^Δ^*^/^*^Δ^* cells infected with lentiviruses expressing either a non-targeting control shRNA (shNTC) or two independent shRNA sequences targeting *Gpt2* (sh*Gpt2*-1; sh*Gpt2*-2). Data are presented as mean ± s.e.m. of *n* = 3 replicates.  ****P*<0.001 (one-way Anova). (**B**) Measurement of LDH activity in media of the indicated cells after 96 hours of culture. Cells treated with 1 mM H_2_O_2_ for 96 hours were used as a positive control of cytotoxicity. Data are presented as mean ± s.e.m. of *n* = 2 replicates per condition of one representative experiment repeated twice. ns: not significant (one-way Anova). (**C**) Average Log2(fold) of depletion of shRNA sequences targeting *GPT* (shGPT) determined in 285 genomically characterized human cancer cell lines retrieved from the Project Achilles plotted in relation to their *SDHB* mRNA levels (microarray) retrieved from Cancer Cell Line Encyclopedia, showing no correlation between sensitivity to *GPT* silencing and *SDHB* hypoexpression. (**D**) Average Log2(fold) of depletion of shRNA sequences targeting *GPT2* (shGPT2) (left) or *GPT* (shGPT) (right) determined in 285 genomically characterized human cancer cell lines retrieved from the Project Achilles plotted in relation to their *SDHC* mRNA levels (microarray) retrieved from Cancer Cell Line Encyclopedia. (**E**) *GPT* and *GPT2* mRNA expression levels (microarray E-MTAB-733) in human pheochromocytoma and paraganglioma tumour specimens with (*SDHx*-mut, n=23) or without (non-*SDHx*-mut, n=165) mutations in genes encoding for succinate dehydrogenase subunits. Each dot indicates one sample, bold black line indicates the mean. ****P*<0.001 (two-tailed Student’s t-test). Data were tested for normality; ns: not significant. (**F**) *GPT* mRNA expression levels (microarray GSE39716) in human pheochromocytoma and paraganglioma tumour specimens with (SDHx-mut, n=32) or without (non-SDHx-mut, n=13) mutations in genes encoding for succinate dehydrogenase subunits and normal adrenal medulla tissue (n= 8). Each dot indicates one sample, bold black line indicates the mean; ns: not significant (one-way Anova). (**G**) Correlation analysis of *GPT2* and *SDHB* mRNA levels in human gastrointestinal stromal tumors (GISTs) (GSE136755) harboring oncogenic mutations in *KIT* or *PDGFRA* genes. *r* = Pearson's correlation coefficient. (**H**) qPCR analysis of *Gpt2* mRNA levels in *Sdhb^fl^*^/^*^fl^* and *Sdhb^Δ^*^/^*^Δ^* cells. Data are presented as mean ± s.e.m. of *n* = 3 replicates.  Top panel, representative western blot analysis of GPT2 and SDHB protein levels in the indicated cells. β-Actin was used as a loading control; kDa (molecular weight). (**I**) qPCR analysis of *Gpt* mRNA levels in *Sdhb^fl^*^/^*^fl^* and *Sdhb^Δ^*^/^*^Δ^* cells. Data are presented as mean ± s.e.m. of *n* = 3 replicates.  (**J**) Number of *Gpt2*-silenced *Sdhb^fl^*^/^*^fl^* and *Sdhb^Δ^*^/^*^Δ^* cells measured after 96 h of culture in Plasmax medium. Data are presented as mean ± s.e.m. of *n* = 4 replicates.  ****P*<0.001 (one-way Anova).

**Supplementary Figure 3. GPT2 activity drives reductive glutamine carboxylation to sustain glycolysis in SDH-deficient cells.** (**A**) Determination of intracellular GSH to GSSG ratio in *Gpt2*-silenced *Sdhb^fl^*^/^*^fl^* and *Sdhb^Δ^*^/^*^Δ^* cells. Data are presented as mean ± s.e.m. of *n* = 4 replicates. (**B**) Number of *Gpt2*-silenced *Sdhb^fl^*^/^*^fl^* and *Sdhb^Δ^*^/^*^Δ^* cells measured after 96 h of culture in the presence/absence of the highest subtoxic concentration of N-acetyl-cysteine (NAC, 0.5 mM) or 0.2 mM trolox in the medium. Data are presented as mean ± s.e.m. of *n* = 4 replicates.  (**C**) Heatmap depicting the levels of the indicated free fatty acids in *Gpt2*-silenced *Sdhb^fl^*^/^*^fl^* and *Sdhb^Δ^*^/^*^Δ^* cells. Data are presented as row normalized Z-scores of peak area/ug proteins of *n* = 4 replicates. (**D**) qPCR analysis of mRNA levels of the indicated genes in *Gpt2*-silenced *Sdhb^fl^*^/^*^fl^* and *Sdhb^Δ^*^/^*^Δ^* cells. Data are presented as mean ± s.e.m. of *n* = 6 replicates. For both genes, the differences between the means are not significant compared with shNTC cells (Brown-Forsythe ANOVA test). Intracellular NAD^+^ to NADH ratio (**E**), glycolytic intermediates (**F**) and ATP (**G**) levels in *Gpt2*-silenced *Sdhb^fl^*^/^*^fl^* and *Sdhb^Δ^*^/^*^Δ^* cells cultured for 24h in the presence of 20 mM aspartate in the medium. Data are presented as mean ± s.e.m. of at least n = 3 replicates. ASP: aspartate; P: phosphate; BP: bisphosphate, PG: phosphoglycerate; Pep: phosphoenolpyruvate.

**Supplementary Figure 4. A metabolic corollary identifies NAD biosynthesis as a targetable vulnerability to SDH loss.** (**A**) qPCR analysis of NAMPT mRNA levels in *Sdhb^fl^*^/^*^fl^* and *Sdhb^Δ^*^/^*^Δ^* cells. Data are presented as mean ± s.e.m. of *n* = 3 replicates. Top panel, representative western blot analysis of NAMPT and SDHB protein levels in the indicated cells. β-Actin was used as a loading control. kDa (molecular weight). (**B**) qPCR analysis of mRNA levels of key enzymes involved in NAD biosynthesis in *Sdhb^fl^*^/^*^fl^* and *Sdhb^Δ^*^/^*^Δ^* cells. Data are presented as mean ± s.e.m. of at least *n* = 3 replicates. **P*<0.05; ***P*<0.01 compared with *Sdhb^fl/fl^* (two-tailed Student’s *t*-test); ns: not significant; nd: not detectable. (**C**) Number of *Sdhb^Δ^*^/^*^Δ^* cells measured after 72 h of culture in the presence/absence of 12 nM FK866, 100 µM NAD^+^ or 100 µM NMN in the medium. Data are presented as mean ± s.e.m. of *n* = 4 replicates.  ****P*<0.001 (two-tailed Student’s *t*-test). (**D**) Number of *Gpt2*-silenced *Sdhb^fl^*^/^*^fl^* cells measured after 5 days of culture in the presence/absence of 3 nM FK866 in the medium. Data are presented as mean ± s.e.m. of *n* = 6 replicates. (**E**) Representative western blot analysis of SDHB protein levels in SDHB-deficient RENCA cells (RENCA^sgSDHB^), generated by CRISPR/Cas9-mediated editing guided by a *Sdhb*-specific single-guide RNA (sgRNA), and SDHB-expressing counterparts (RENCA^sgNTC^), generated by using a non-targeting control (NTC) sgRNA. β-Actin was used as a loading control. kDa (molecular weight). (**F**) Number of RENCA^sgNTC^ RENCA^sgSDHB^ cells measured after 96 h of culture in the presence/absence of 25 nM FK866 in the medium. Data are presented as mean ± s.e.m. of at least *n* = 9 replicates ****P*<0.001 (two-tailed Student’s *t*-test). (**G**) Determination of NAD^+^ in tumours isolated from mice treated as in Fig. 4H. Data are presented as mean ± s.e.m. **P*<0.05 compared with FK866-treated group; #*P*<0.05 compared with FK866 + α-TOS-treated group (Brown-Forsythe ANOVA test followed by Dunnett's T3 multiple comparisons test). Potential outliers were identified by the ROUT method according to GraphPad guidelines and removed from experimental groups for more accurate calculation of statistical difference among means. (**H**) Representative images of Ki-67 (top panels) and cleaved-Caspase 3 (bottom panels) immunohistochemical staining in tumours isolated from mice treated as in Fig. 4H. Bar = 200 μm.
