# Supplementary figures and images for: Pyruvate transamination and NAD biosynthesis enable proliferation of succinate dehydrogenase-deficient cells by supporting aerobic glycolysis
Source: Cell Death Dis. 2023 Jul 6;14(7):403. doi: 10.1038/s41419-023-05927-5 (PMC10326256; doi:10.1038/s41419-023-05927-5)

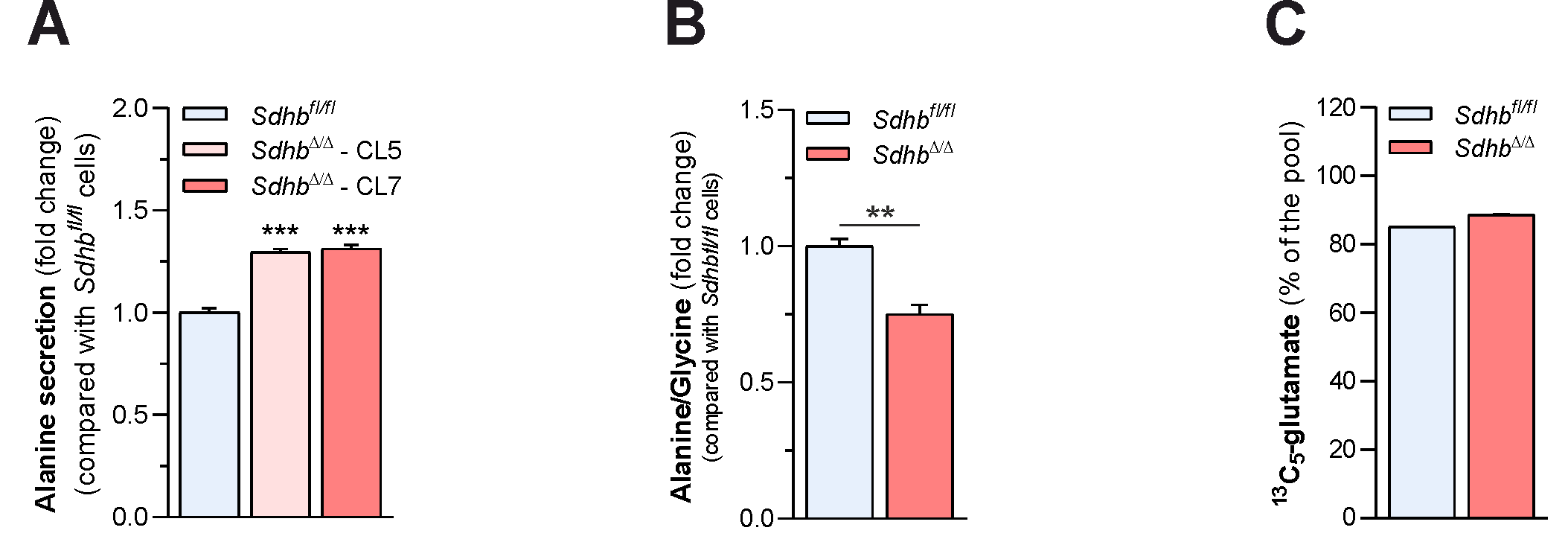

Supplement: Supplementary file 2 — Supplementary Figure 1 [file 41419_2023_5927_MOESM2_ESM.tif]

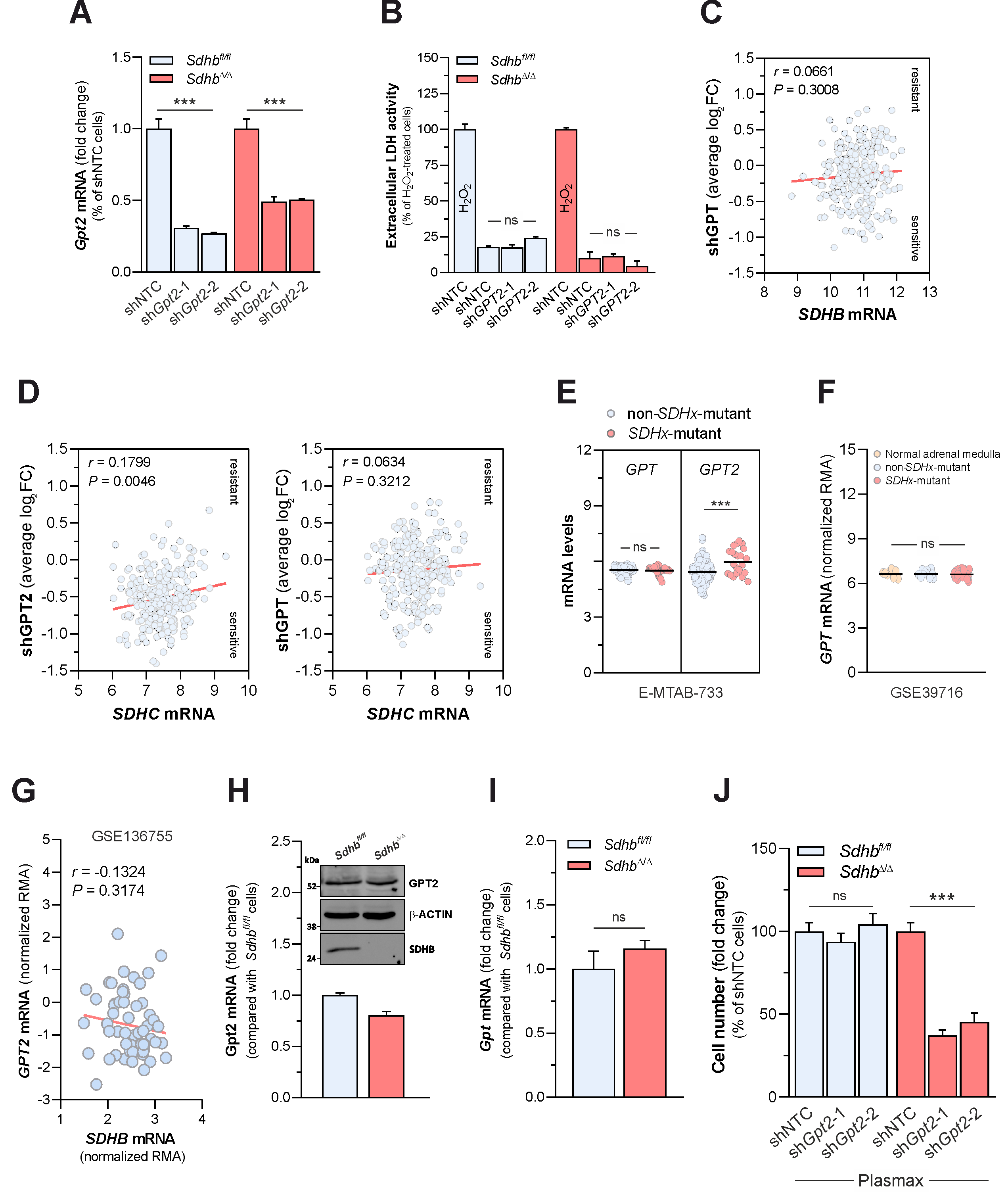

Supplement: Supplementary file 3 — Supplementary Figure 2 [file 41419_2023_5927_MOESM3_ESM.tif]

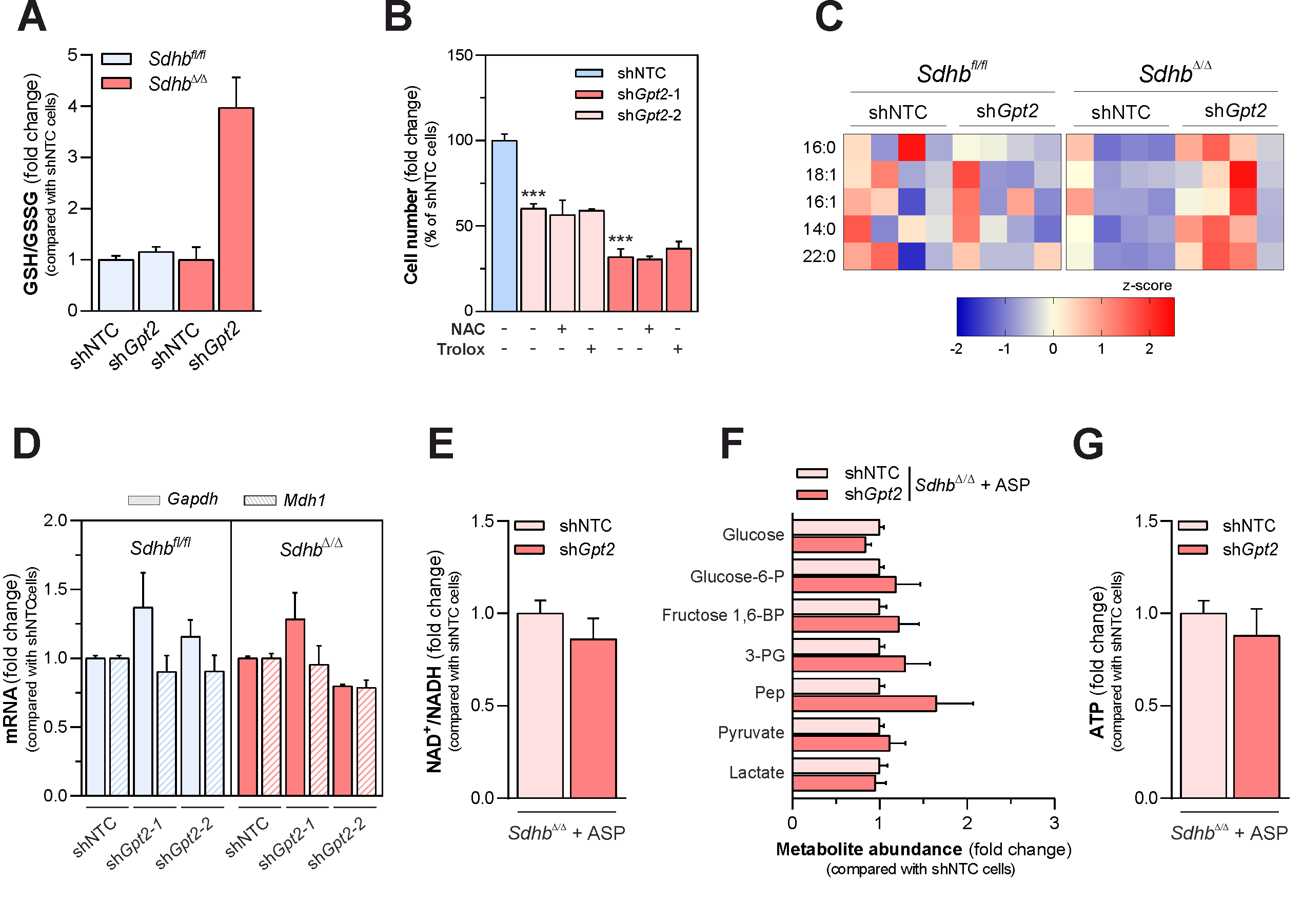

Supplement: Supplementary file 4 — Supplementary Figure 3 [file 41419_2023_5927_MOESM4_ESM.tif]

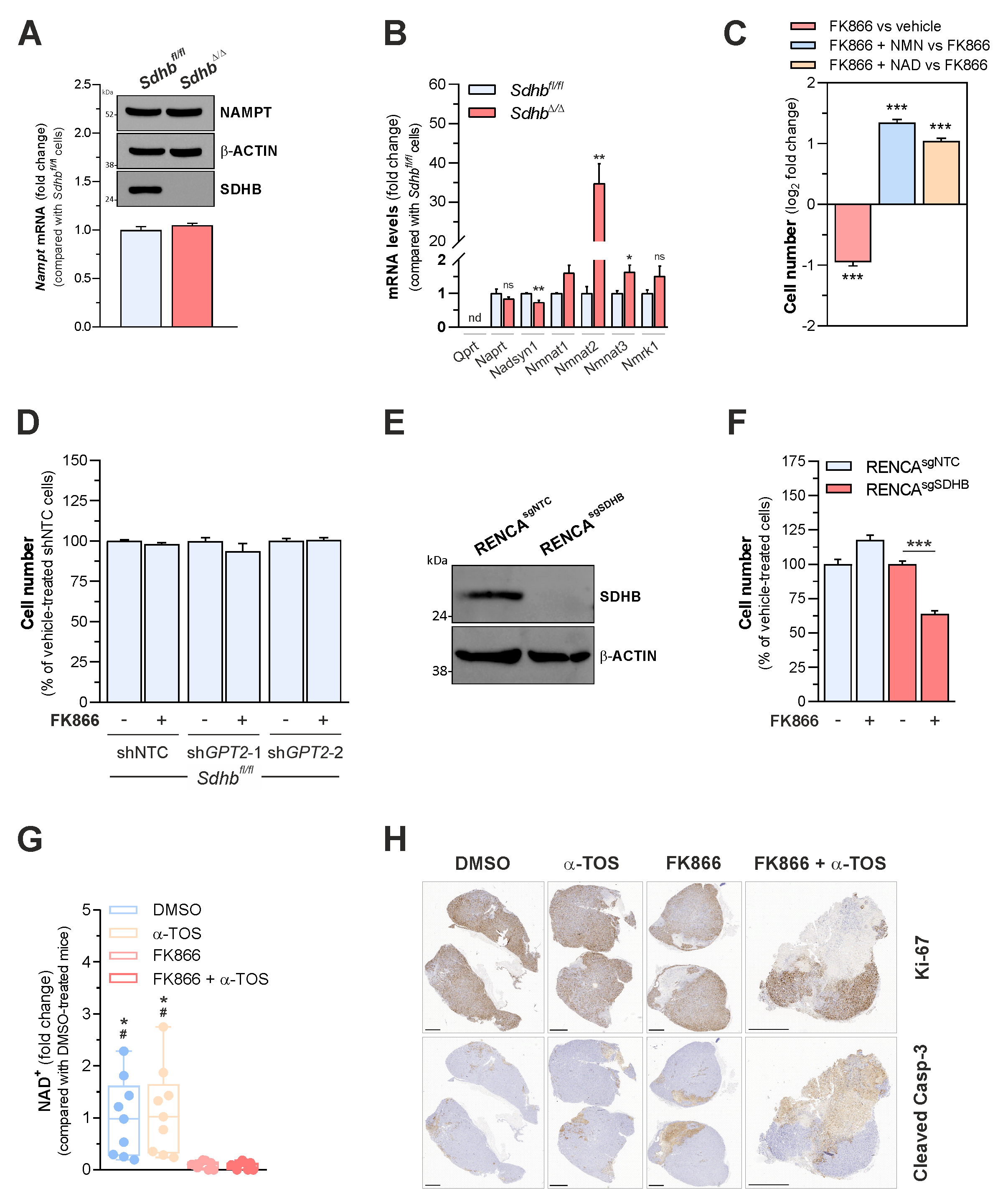

Supplement: Supplementary file 5 — Supplementary Figure 4 [file 41419_2023_5927_MOESM5_ESM.tif]

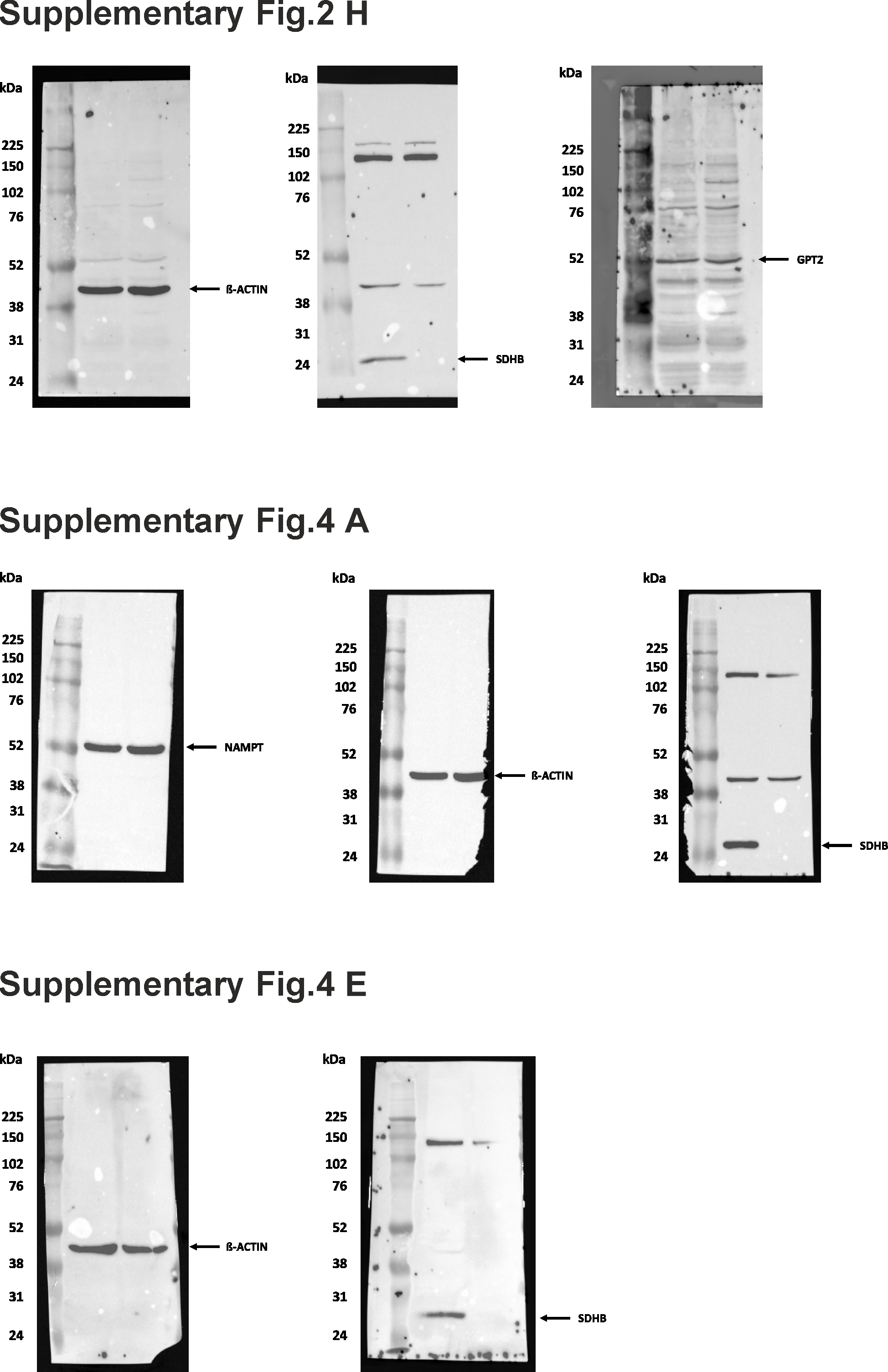

Supplement: Supplementary file 6 — Supplementary Material - Uncropped Western Blots [file 41419_2023_5927_MOESM6_ESM.tif]
